# Fat extract promotes angiogenesis in a murine model of limb ischemia: a novel cell-free therapeutic strategy

**DOI:** 10.1186/s13287-018-1014-y

**Published:** 2018-11-08

**Authors:** Ziyou Yu, Yizuo Cai, Mingwu Deng, Dong Li, Xiangsheng Wang, Hongjie Zheng, Yuda Xu, Wei Li, Wenjie Zhang

**Affiliations:** Department of Plastic and Reconstructive Surgery, Shanghai Key Laboratory of Tissue Engineering, Shanghai 9th People’s Hospital, Shanghai Jiao Tong University, 639 Zhi Zao Ju Road, Shanghai, 200011 China

**Keywords:** Fat extract, Adipose tissue stromal/stem cells, Growth factors, Angiogenesis, Critical limb ischemia, Regeneration

## Abstract

**Background:**

The proangiogenic capacity of adipose tissue and its derivatives has been demonstrated in a variety of studies. The paracrine mechanism of the cellular component is considered to play a critical role in the regenerative properties of these tissues. However, cell-based therapy for clinical application has been hindered by limitations such as safety, immunogenicity issues, and difficulties in cell preservation, transportation, and phenotype control. In the current study, we aimed to produce a cell-free extract directly from human fat tissue and evaluate its potential therapeutic efficacy.

**Methods:**

We developed a novel physical approach to produce a cell-free aqueous extract from human fat tissue (fat extract (FE)). The therapeutic potential of FE was investigated in the ischemic hindlimb model of nude mice. After establishment of hindlimb ischemia with ligation of the left femoral artery and intramuscular injection of FE, blood perfusion was monitored at days 0, 7, 14, 21, and 28. Tissue necrosis and capillary density were evaluated. Enzyme-linked immunosorbent assay was used to analyze the growth factors contained in FE. Moreover, the proliferation, migration, and tube formation ability were tested on human umbilical vein endothelial cells (HUVECs) in vitro when treated with FE. The proangiogenic ability of FE was further assessed in an in-vivo Matrigel plug assay.

**Results:**

FE was prepared and characterized. The intramuscular injection of FE into the ischemic hindlimb of mice attenuated severe limb loss and increased blood flow and capillary density of the ischemic tissue. Enzyme-linked immunosorbent assay showed that FE contained high levels of various growth factors. When added as a cell culture supplement, FE promoted HUVEC proliferation, migration, and tube formation ability in a dose-dependent manner. The subcutaneous injection of Matrigel infused with FE enhanced vascular formation.

**Conclusions:**

We developed a novel cell-free therapeutic agent, FE, produced from human adipose tissue. FE was able to attenuate ischemic injury and stimulate angiogenesis in ischemic tissues. This study indicates that FE may represent a novel cell-free therapeutic agent in the treatment of ischemic disorders.

## Background

Therapeutic angiogenesis is a promising approach for the treatment of cardiovascular diseases. In recent years, stem cell therapy has emerged as a promising therapeutic strategy for ischemic conditions. Among the cell types under investigation, adipose tissue-derived stromal/stem cells (ADSCs) have attracted great attention owing to their ease of isolation, relative abundance, stable capacity to self-renew, multipotent differentiation capacity, and low immunogenicity [1–3]. A great number of animal studies and clinical trials involving ADSCs have demonstrated their ability to promote angiogenesis in ischemic heart and cerebral diseases, peripheral vascular disease, and chronic wounds, among other conditions [4–7]. Further studies have revealed that the therapeutic effects of ADSCs occur not primarily through in-situ differentiation but via the secretion of growth factors [8, 9]. ADSCs express a broad spectrum of paracrine factors that are known to be angiogenic, such as vascular endothelial growth factor (VEGF), basic fibroblast growth factor (bFGF), interleukin 6 (IL-6), and transforming growth factor alpha (TGF-α) [10]. Subsequent studies have confirmed that the administration of ADSC-conditioned medium (ADSC-CM) could also have efficient therapeutic effects on ischemic diseases [11, 12]. Thus, instead of cell transplantation, the delivery of ADSC-secreted factors has recently been considered an alternative strategy, as it can circumvent many safety concerns and limitations related to the use of cultured cells. However, the collection of ADSC-CM or its secretome for clinical application is still complicated and time consuming. Meanwhile, the expensive cell manufacturing process and functional control remain to be overcome.

Adipose tissue has currently gained significant importance since it serves as an abundant source of ADSCs. As a result, adipose tissue and its derivatives, initially used for soft tissue augmentation, have now been used for regenerative purposes. For example, the autologous fat graft has been used to successfully treat irradiated tissue and chronic ischemia [13–17]. The stromal vascular fraction (SVF), a mixed cell population commonly isolated by the enzymatic digestion of fat, has been used in therapies for burn injury and diabetes [18, 19]. More recently, nanofat, a fat emulsion produced through mechanical forces, has been shown to improve fat graft survival and skin rejuvenation and has been used in the treatment of atrophic scars, among other conditions [20–25]. The regenerative effects of the aforementioned materials are considered mainly related to their cellular component via the secretion of growth factors. Interestingly, Sarkanen et al. [26] demonstrated that adipose tissue itself secretes large amounts of growth factors. After the incubation of adipose tissue for 24 h, the tissue culture medium contains numerous growth factors and cytokines that promote angiogenesis and adipogenesis in vitro and in vivo. Pallua et al. [27] found that fresh lipoaspirate contained a certain amount of proangiogenic factors, such as bFGF, VEGF, and platelet-derived growth factor (PDGF). These results indicate that adipose tissue is inherently enriched with a variety of bioactive factors that might be directly isolated for clinical application without the cell isolation or cultivation process.

Our previous work found that nanofat contains a certain amount of angiogenic factors, including VEGF, bFGF, PDGF, hepatocyte growth factor (HGF), granulocyte–macrophage colony-stimulating factor (GM-CSF), transforming growth factor beta (TGF-β), and insulin-like growth factor 1 (IGF-1) [28]. The cotransplantation of nanofat enhances fat graft survival in nude mice by promoting angiogenesis [28]. The subcutaneous injection of nanofat increases dermal neovascularization in a photoaging skin model [24]. Studies have also shown that, when centrifuged, nanofat consists of an oil layer, a cellular/extracellular matrix fraction, and a liquid fraction [29–31]. The cellular/extracellular matrix fraction, known as “SVF-gel”, is composed of a high concentration of ADSCs and a functional extracellular matrix that could promote angiogenesis in ischemic tissues [29, 30, 32]. Unfortunately, the liquid fraction has been ignored in previous studies. If the liquid portion contains a high level of growth factors, it might also possess therapeutic activity. More importantly, use of the cell-free liquid fraction could theoretically avoid the cell-related concerns in clinical applications; these concerns include the genetic stability of cells after processing, cell activity and survival after injection, and the storage of cells for multiple administration, as well as the immunogenicity of cells when using allogeneic fats. Based on these clues, we purified the liquid fraction, namely the “fat extract” (FE), from nanofat using a mechanical approach to remove the cellular components and the lipid remnants. We speculate that the cell-free aqueous component derived from fat may possess a similar proangiogenic function and exhibit therapeutic potential in reducing ischemic injury.

In the present study, we first developed a novel physical approach to isolate the cell-free aqueous component from human fat and then evaluated its therapeutic effects in the hindlimb ischemic model of nude mouse. Moreover, to explore the underlying mechanisms of FE on ischemic limb angiogenesis, the angiogenic factors within FE were further measured and the proangiogenic capacity of FE was evaluated on cultured human umbilical vein endothelial cells (HUVECs) in vitro as well as on the murine Matrigel plug assay in vivo.

## Methods

### FE preparation

Human liposuction aspirates were obtained from six healthy female donors who underwent liposuction from October 2017 to April 2018 in Shanghai 9th People’s Hospital, Shanghai, China after providing written informed consent. The mean age was 31 years (range 24–36 years). The study was approved by the Ethics Committee of Shanghai Jiaotong University School of Medicine, Shanghai, China. A standard traditional liposuction cannula with large side holes (2 mm × 7 mm) was used to harvest the macrofat, as previously described [20].

**Fig. 1:**
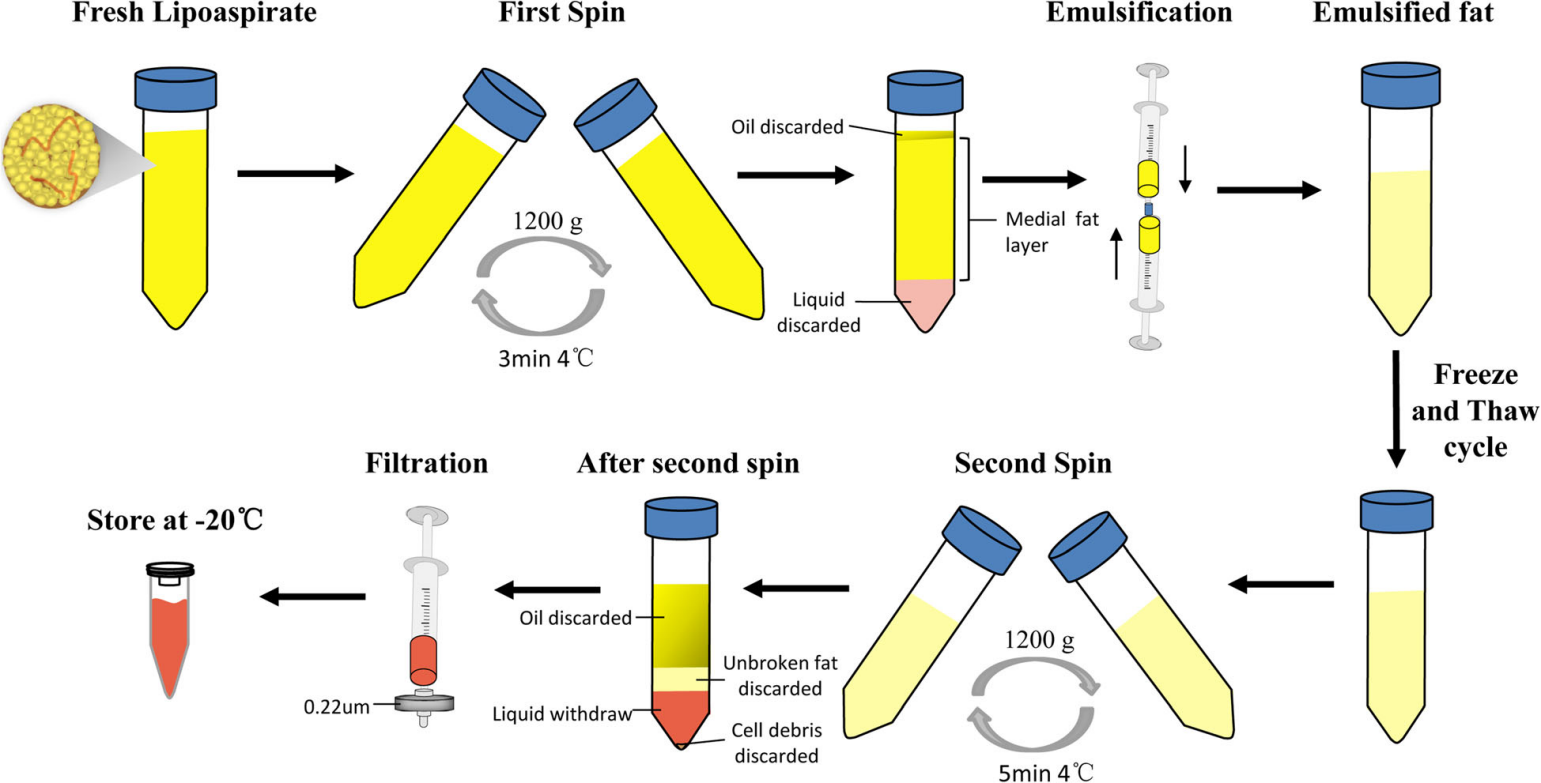
Schematic illustration of FE preparation

The detailed procedures for isolating FE are shown in Fig. 1. The lipoaspirate was first rinsed with saline to remove red blood cells and then centrifuged at 1200 × *g* for 3 min. After the first spin, the superior oily and inferior fluid layers were discarded, and the middle fat layer was collected and mechanically emulsified. The emulsification was achieved via 30 passes of shifting the fat between two 10-cm^3^ syringes connected by a female-to-female Luer-Lok connector (B. Braun Medical Inc., Melsungen, Germany). The emulsified fat was then frozen at − 80 °C and thawed at 37 °C for further disruption of the fat tissue. After one cycle of the freeze/thaw process, the fat was again centrifuged at 1200 × *g* for 5 min. After a second spin, the fat was separated into four layers. The upper layer of oil was discarded; the second layer of unbroken fat and the fourth layer of debris was discarded; and the third aqueous layer, namely the FE, was carefully aspirated without contamination of the bottom pellet. The final extract was produced by passing it through a 0.22-μm filter (Corning Glass Works, Corning, NY, USA) for sterilization and removal of cell debris. The extract was then stored at − 20 °C for future use. The protein concentrations of FE were measured with a Pierce BCA protein assay kit (Thermofisher Scientific, Waltham, MA, USA).

### Hindlimb ischemia model

A unilateral hindlimb ischemia model was generated in nude mice, aged 10–12 weeks, via ligation of the left femoral artery and its branches, as previously described [33, 34]. In brief, the mice were anesthetized via isoflurane (2–3%) inhalation. The femoral artery was isolated from the femoral nerve and vein, and then ligated and excised below the inguinal ligament and above the bifurcation of the popliteal artery. Two doses of FE (50 μl for the FE^Low^ group and 100 μl for the FE^High^ group, approximately 232.27 μg and 474.54 μg of protein, respectively) or 100 μl of PBS for the control group (*n* = 10 per group) were injected intramuscularly immediately after surgery and again at 24 h post operation. All animals were monitored daily and sacrificed at 28 days post operation. The gastrocnemius muscle of the calf was harvested from the ischemic limbs for histological analysis and capillary density evaluation.

### Blood flow analysis and tissue necrosis scoring

The laser Doppler perfusion imaging (LDPI) analyzer (Moor Instruments, Axminster, UK) was used to record blood flow measurements on days 0, 7, 14, 21, and 28 post operation. Blood perfusion of the calf region (from ankle to the knee) of both limbs was selected as the regions of interest to facilitate LDPI analysis. The recovery of perfusion was calculated as the ratio of ischemic hindlimb blood perfusion to nonischemic hindlimb blood perfusion. A ratio of 1 before surgery indicated equal blood perfusion of both legs. Tissue necrosis was scored as previously described [34]: 0, normal/no necrosis; 1, mild necrosis and/or deep cyanosis of toes; 2, necrosis/amputation of toes (two or more); 3, foot amputation; and 4, severe limb loss.

### Histological evaluation

For histological analysis, the gastrocnemius muscles were fixed in 4% paraformaldehyde for 24 h, embedded in paraffin, cut into sections 5 μm thick, and stained with hematoxylin–eosin (HE). The percentage of tissue necrosis was quantified using Image Pro Plus 6.0 software (Media Cybernetics, Silver Spring, MD, USA). For the evaluation of the capillary density, the specimens were incubated in rabbit monoclonal anti-CD31 antibody (ab182981; Abcam, Cambridge, UK) overnight at 4 °C. HRP-labeled polymer anti-rabbit antibody (Dako, Glostrup, Denmark) was then added for 30 min at 37 °C. A 3,3′-diaminobenzidine substrate kit (Boster, Wuhan, China) was used to visualize the reaction. For the detection of infiltrated monocytes/macrophages, sections were stained using rabbit anti-mouse CD68 (Servicebio, Wuhan, China). Whole slide images were obtained using a Pannoramic MIDI slide scanner (3DHISTECH Kft., Budapest, Hungary). The capillary density was calculated as the total number of vessels identified in the total section area. The level of monocytes/macrophages infiltration was defined as CD68^+^ cells/mm^2^. Three sections from each specimen were used for analysis.

### Enzyme-linked immunosorbent assay

Enzyme-linked immunosorbent assays (ELISAs) were conducted according to standard protocols to quantify the levels of brain-derived neurotrophic factor (BDNF), glial cell-derived neurotrophic factor (GDNF), TGF-β, HGF, bFGF, VEGF, PDGF, epidermal growth factor (EGF), neurotrophin-3 (NT-3), and granulocyte colony-stimulating factor (G-CSF) in FE.

### Proteomics analysis

Three samples of FE were processed for quantitative proteomic analysis by Jingjie PTM BioLab (Hangzhou, China). Protein concentrations of FE were measured with the BCA protein assay kit. Samples were then digested by trypsin for subsequent LC-MS/MS analysis. The tryptic peptides were fractionated and subjected to neutral spray ionization source followed by tandem mass spectrometry (MS/MS) in Q Exactive™ Plus (Thermo) coupled online to the UPLC. Intact peptides were detected in the Orbitrap at a resolution of 70,000. Peptides were then selected for MS/MS using an NCE setting of 28 and the fragments were detected in the Orbitrap at a resolution of 17,500.

The resulting MS/MS data were processed using the Maxquant search engine (v.1.5.2.8). Tandem mass spectra were identified according to the SwissProt human database concatenated with a reverse decoy database. The mass tolerance for precursor ions was set at 20 ppm in the first search and at 5 ppm in the main search, and the mass tolerance for fragment ions was set at 0.02 Da. Carbamidomethylation on Cys was specified as a fixed modification, and oxidation on Met was specified as a variable modification. The label-free quantification method was LFQ, the FDR was adjusted to < 1%, and the minimum score for peptides was set at > 40.

Gene Ontology (GO) analysis was performed to classify all identified proteins into three categories (cell component, molecular function, and biological process) using the UniPort-GOA database (http://www.ebi.ac.uk/GOA/), InterProScan (http://www.ebi.ac.uk/interpro/), and GO annotation (http://geneontology.org/). Proteins related to angiogenesis were identified.

### Cell culture

HUVECs, purchased from the American Type Culture Collection (ATCC, Rockville, MD, USA), were cultured in DMEM supplemented with 10% fetal bovine serum and 1% penicillin–streptomycin antibiotic. Cultures were maintained in a humidified atmosphere of 5% CO_2_ and 95% air at 37 °C. The medium was replaced every 2 days, and cell passaging was performed when the monolayer of adherent cells reached 90% confluence.

### Cell proliferation assay

The HUVECs were seeded in a 96-well plate at 2 × 10^3^ cells per well and maintained in DMEM medium containing 10% FBS. After 24 h, the cells were treated with different concentrations of FE (0, 1, 2, and 5% (v/v)) for 3 days. Cells without FE treatment served as controls. The Cell Counting Kit-8 (CCK-8; Dojindo Molecular Technologies, Rockville, MD, USA) was used to evaluate cell proliferation. An absorbance spectrum at 450 nm was recorded using a microplate reader (SpectraMAX i3x; Molecular Devices, Sunnyvale, CA, USA). The data are presented as the ratio of the O.D. value relative to the control group.

### Cell migration assay

The HUVECs were seeded in six-well plates and grown in DMEM medium containing 10% FBS. Upon 90% confluency, the cell monolayer was scratched with a sterile 200-μl pipette tip, washed, and then incubated with serum-free DMEM medium in the presence of FE (0, 1, 2, and 5% (v/v)) for 24 h. VEGF-A 20 ng/ml (Peprotech, Rocky Hill, NJ, USA) was used as a positive control. Images were captured with a digital camera at 0 and 24 h and were measured using ImageJ software (NIH, Bethesda, MD, USA).

### Tube formation assay

The HUVECs (2 × 10^4^ cells per well) were suspended in 100 μl of serum-free medium containing FE (0, 1, 2, and 5% (v/v)) or VEGF-A which acted as a positive control, and then cells were plated under 50 μl of growth factor-reduced Matrigel (BD Biosciences) in 96-well plates after being solidified at 37 °C for 30 min. After incubating in 5% CO_2_ at 37 °C for 6 h, the cells were stained with Calcein-AM solution (Yeason, Shanghai, China) and imaged under fluorescence microscopy (Carl Zeiss, Oberkochen, Germany). The number of branch points per mm^2^ and the mean tube length were measured using ImageJ software (NIH).

### In-vivo Matrigel plug assay

Male BALB/c nude mice (6 weeks old) were purchased from the Animal Laboratory, Shanghai 9th People’s Hospital, Shanghai Jiaotong University School of Medicine, Shanghai, China. The study protocols were approved by The Animal Care and Experiment Committee of the Shanghai Jiaotong University School of Medicine. Twelve mice were randomly divided into three groups (*n* = 4 in each group): the control group, the FE^High^ group, and the FE^Low^ group. The mice in the control group were injected with a mixture of 250 μl of Matrigel and 250 μl of phosphate-buffered saline (PBS); the mice in the FE^Low^ group were injected with a mixture of 250 μl of Matrigel, 125 μl of FE, and 125 μl of PBS; and the mice in the FE^High^ group were injected with a mixture of 250 μl of Matrigel and 250 μl of FE. A total of 500 μl of mixture per plug was injected subcutaneously into the dorsal region of the mice. Seven days after implantation, the Matrigel plugs were harvested for histological analysis. The CD31^+^ capillary density as well as the level of CD68^+^ monocytes/macrophages infiltration was evaluated as already described.

### Statistical analyses

The results are expressed as the mean ± standard deviation. Differences between groups were evaluated using one-way analyses of variance (SPSS Inc., Chicago, IL, USA). All tests were two-sided, with a significance level of *p* < 0.05.

## Results

### Extraction of FE

FE from the lipoaspirate of six healthy volunteers was prepared separately according to the protocol described earlier (Fig. 1). Approximately 7 ml of pinkish FE was finally achieved from 50 ml of centrifuged lipoaspirate (collected after the first spin). The total protein concentration of FE was 4745.43 ± 751.73 μg/ml (*n* = 6).

### FE attenuated tissue necrosis in a hindlimb ischemia mouse model

**Fig. 2:**
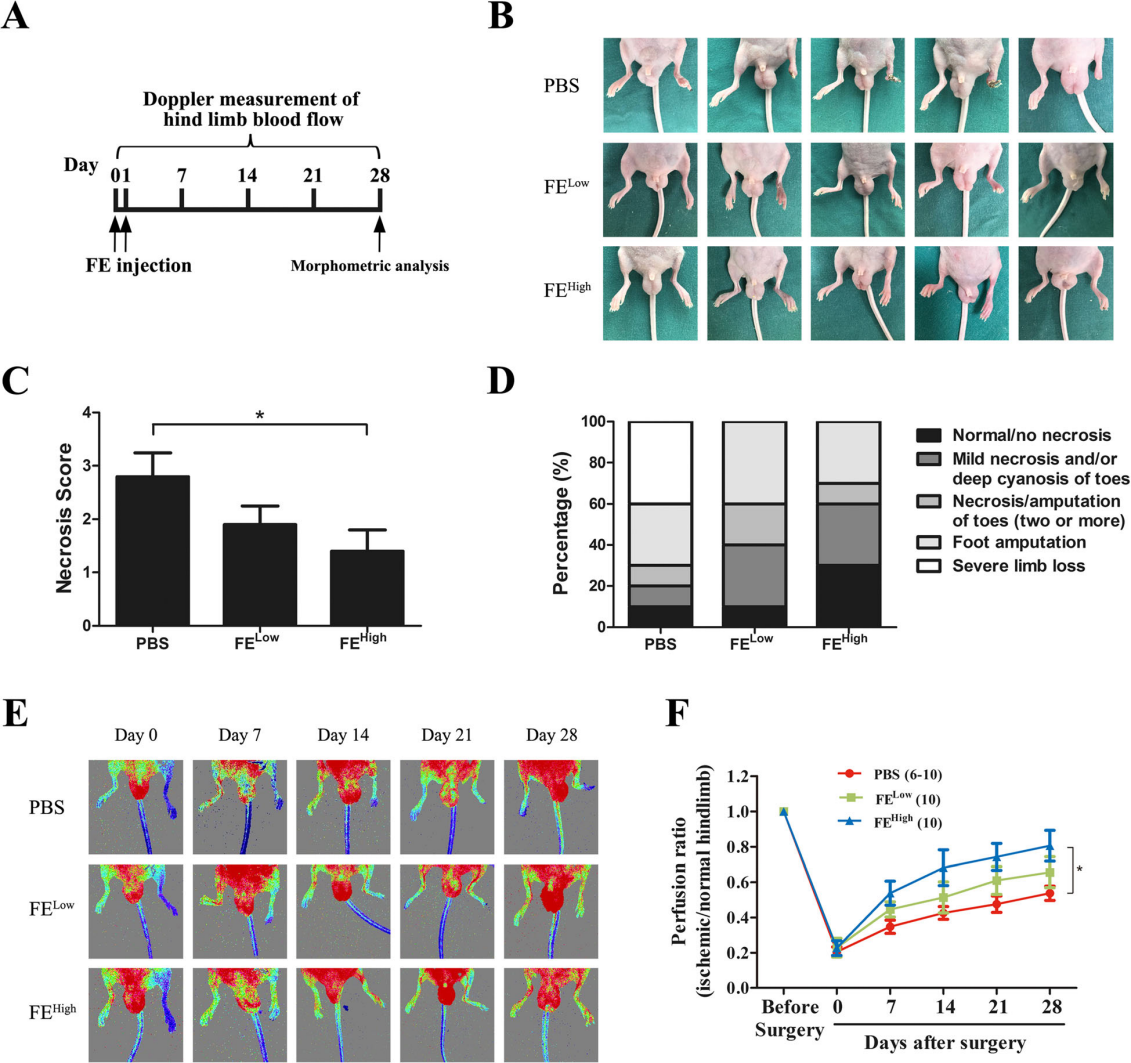
Therapeutic efficacy of FE on limb salvage and blood perfusion in nude mice after critical limb ischemia. **a** Flowchart for treatment of hindlimb ischemia and evaluation methods. **b** Representative images of gross observation at 28 days post operation. **c** Scores of tissue damage in FE^High^ group were significantly decreased at day 28. **d** Ratio of limb outcomes demonstrates improved limb salvage in FE^High^ group. **e** Laser Doppler perfusion imaging (LDPI) showed dynamic changes in blood perfusion in limb ischemia of each group at days 0, 7, 14, 21, and 28 post operation. **f** Quantitative analysis of blood flow measured by LDPI. Ratio of LDPI in ischemic hindlimb to contralateral hindlimb calculated over observation period. Data represent mean ± SD (*n* = 10 per group). *p < 0.05. FE fat extract, PBS phosphate-buffered saline

To first evaluate the therapeutic effects of FE on ischemic disorders, the rescue ability of FE in a murine model of hindlimb ischemia was tested. Because rapid revascularization at the very early stage of ischemia is crucial for functional restoration of the ischemic tissue, FE was injected into the ischemic hindlimbs immediately after surgery and 24 h later (Fig. 2a). By day 28, the FE injection resulted in significantly attenuated tissue necrosis in the groups receiving low and high doses of FE treatment compared with that in the group that received PBS treatment (Fig. 2b–d). Blood flow at the calf level was measured by LDPI on days 0, 7, 14, 21, and 28 post operation. Four mice in the PBS group developed severe limb necrosis and were excluded from LDPI measurements, reducing the numbers of animals available for analysis from 10 to 6 over time. LDPI showed that blood flow recovered quickly in the FE-treated groups but not in the control group (Fig. 2e). By day 28, 80.59% and 65.60% of blood flow was recovered in the groups that received high and low doses of FE, respectively; both of these results were higher than that in the PBS-treated group (53.71%). A significant difference was observed between the FE^High^-treated group and the control group (Fig. 2f).

**Fig. 3:**
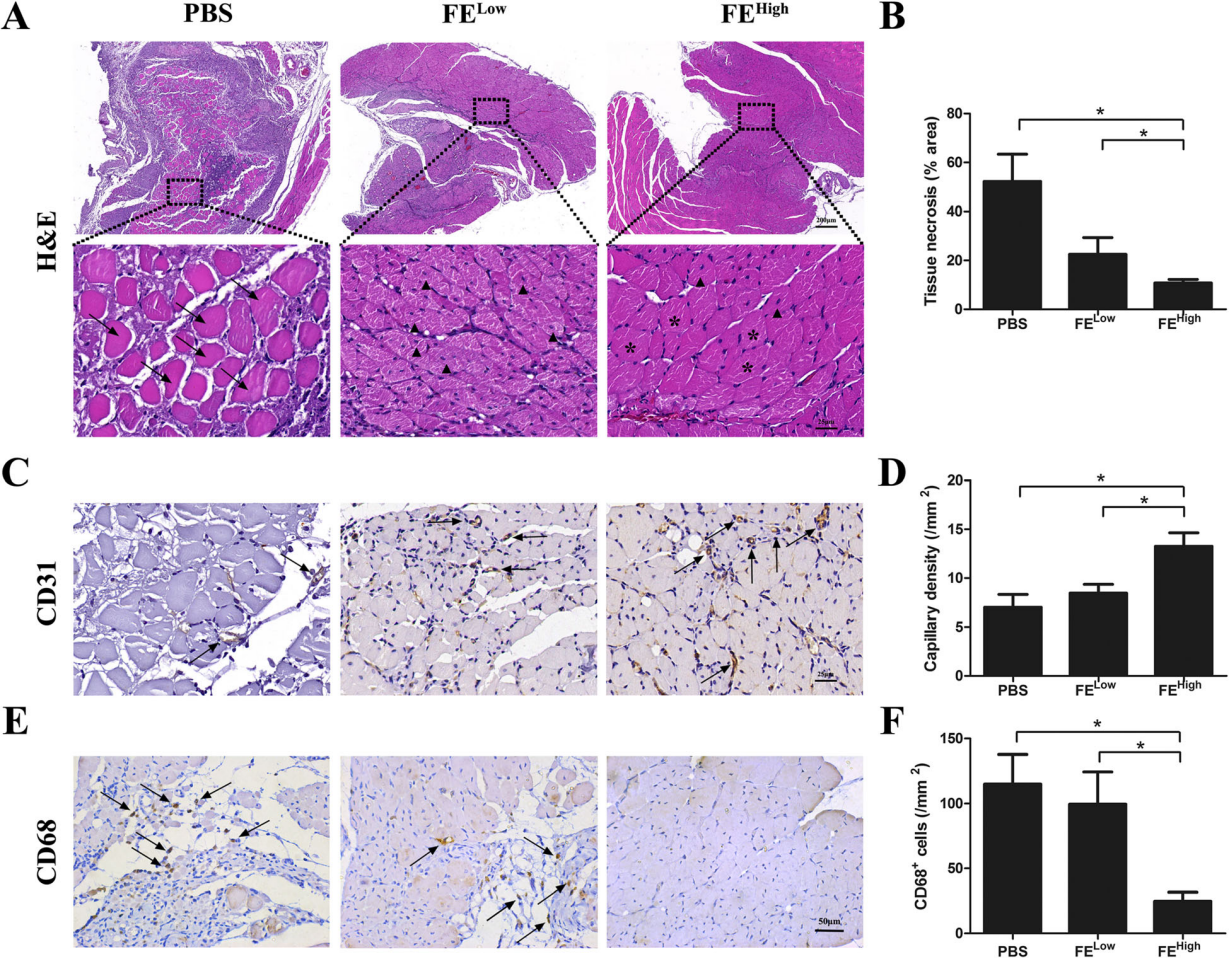
Histological evaluation of necrosis and angiogenesis in ischemic hindlimb**. a** Hematoxylin–eosin (H&E) staining of gastrocnemius (GC) muscle of calf. Arrows indicate necrotic myofibers; arrowheads indicate regenerating myofibers characterized by central nuclei; asterisks indicate healthy myofibers with peripheral nuclei. Although H&E staining showed muscle degeneration and necrosis in all ischemic limbs, a higher degree of muscle degeneration and necrosis was observed in PBS-treated group. **b** Quantitative analysis of percentage area of necrotic tissue. Significantly fewer percentage areas of necrotic tissues measured in FE^High^-treated group. **c** Immunostaining of CD31^+^ capillaries in ischemic GC muscles. Arrows indicate blood vessels. FE-treated group showed more CD31^+^ blood vessels. **d** Quantitative analysis of capillary density. Significantly higher blood vessel density was measured in FE^High^-treated group. **e** Immunostaining of CD68 in ischemic GC muscles. Arrows indicate CD68^+^ inflammatory cells. **f** Quantification of CD68^+^ cell density. FE^High^-treated group showed significantly lower CD68^+^ inflammatory cell infiltration. *p < 0.05. FE fat extract, PBS phosphate-buffered saline

The mice were sacrificed on day 28, and the gastrocnemius muscles of the ischemic limbs were harvested for histological analysis. Hematoxylin–eosin staining of the muscle showed that the muscle in the control group had massive degeneration and necrosis, whereas muscle degeneration and necrosis of the ischemic limb was largely protected in the FE-treated group (Fig. 3a). Quantitative analysis revealed a lower percentage area of necrotic tissue in the FE-treated group as compared to the control group (Fig. 3b). To identify blood vessels, immunohistochemical staining was performed to assess the CD31^+^ capillaries in the ischemic muscles. More CD31^+^ capillaries were observed in the FE-treated groups compared with those in the control group (Fig. 3c). The densities of the capillaries were significantly higher in the FE-treated groups than those in the PBS-treated group (Fig. 3d). The increased numbers of CD31^+^ capillaries correlated with the increased blood perfusion measured by laser Doppler in the FE-treated groups. To evaluate inflammatory cell infiltration, sections of gastrocnemius muscles were stained for CD68 (Fig. 3e). The densities of CD68^+^ cells were significantly lower in the FE-treated group compared with the control group (Fig. 3f). These results indicate that FE attenuated tissue necrosis in ischemic hindlimbs likely through accelerating neovascularization.

### Growth factor content and individual variation in growth factor concentrations in FE

**Fig. 4:**
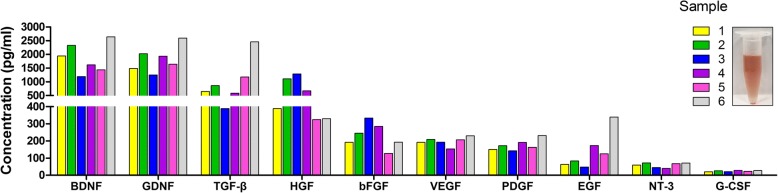
Interindividual distribution of growth factor levels in FE. BDNF brain-derived neurotrophic factor, GDNF glial cell-derived neurotrophic factor, TGF-β transforming growth factor beta, HGF hepatocyte growth factor, bFGF basic fibroblast growth factor, VEGF vascular endothelial growth factor, PDGF platelet-derived growth factor, EGF epidermal growth factor, NT-3 neurotrophin-3, G-CSF granulocyte–macrophage colony-stimulating factor

To verify the underlying mechanism of FE treatment, the angiogenic factors within FE were then measured in six samples using ELISA. High levels of growth factors, including BDNF, GDNF, TGF-β, HGF, bFGF, VEGF, PDGF, EGF, NT-3, and G-CSF, were detected in the FE (Fig. 4). The mean level and variation of each factor in the six samples are presented in Table 1.

**Table 1:** Mean fat extract growth factor concentrations (pg/ml)

	BDNF	GDNF	TGF-β	HGF	bFGF	VEGF	PDGF	EGF	NT-3	G-CSF
Mean	1860.99	1823.23	1019.72	685.47	229.26	197.27	175.26	138.66	59.16	23.98
SD	503.23	433.31	687.48	384.38	67.51	23.50	29.78	98.80	12.40	3.14

*BDNF* brain-derived neurotrophic factor, *GDNF* glial cell-derived neurotrophic factor, *TGF-β* transforming growth factor beta, *HGF* hepatocyte growth factor, *bFGF* basic fibroblast growth factor, *VEGF* vascular endothelial growth factor, *PDGF* platelet-derived growth factor, *EGF* epidermal growth factor, *NT-3* neurotrophin-3, *G-CSF* granulocyte–macrophage colony-stimulating factor, *SD* standard deviation

### Proteomic data analysis: Gene Ontology classification of the quantified proteins

**Fig. 5:**
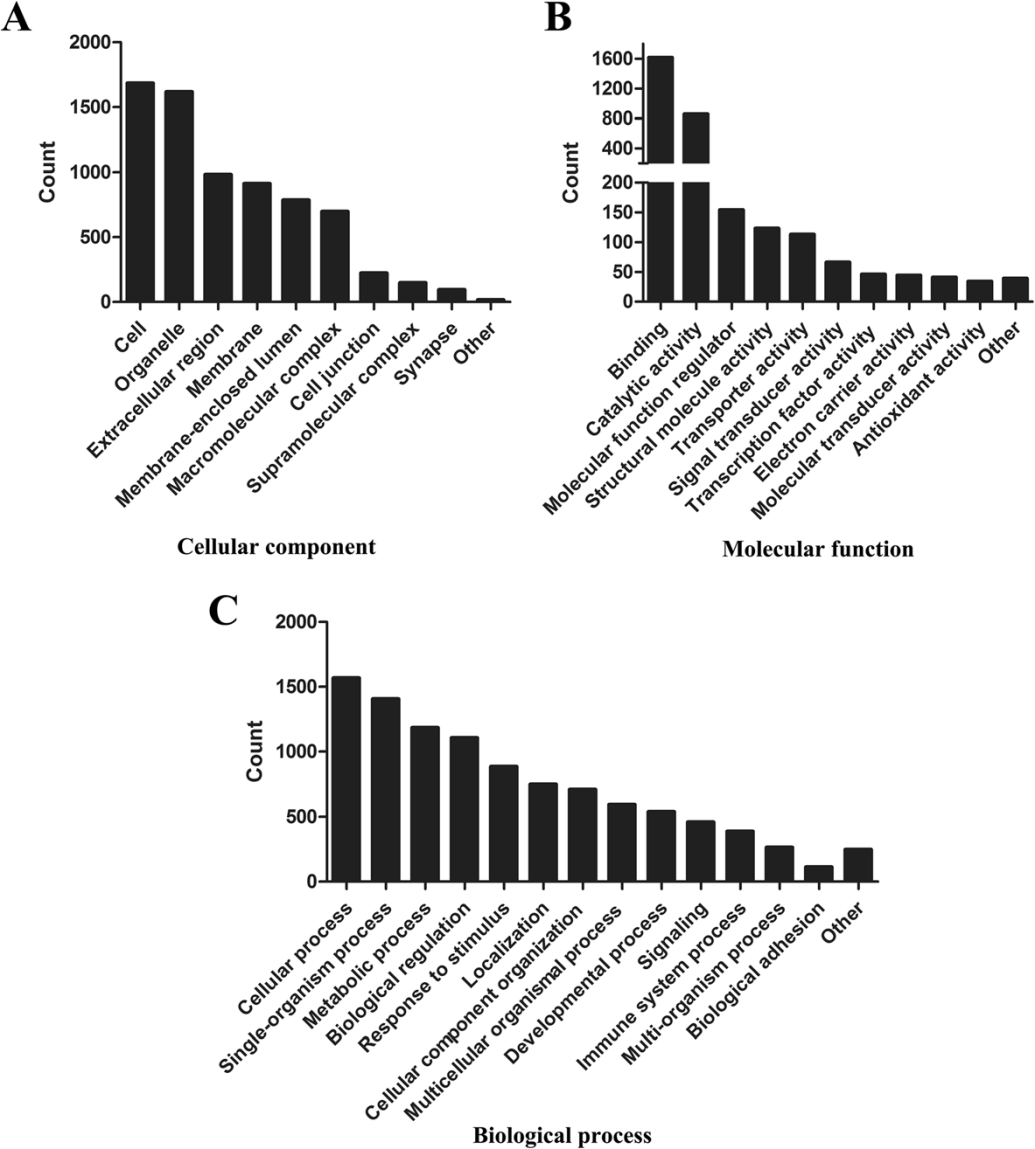
Classification of identified proteins based on Gene Ontology terms. Identified proteins classified into (**a**) cellular component, (**b**) molecular function, and (**c**) biological process using Gene Ontology annotation

The protein composition of FE was determined using mass spectrometry technology. A total of 1767 proteins were identified in all three samples. Proteins were classified by Gene Ontology (GO) annotation based on three categories: cellular component, molecular function, and biological process (Fig. 5). For the cellular component, most of the quantified proteins were in the cell, organelle, and extracellular region GO category (Fig. 5a). The molecular functional category of the majority proteins included binding, catalytic activity, and molecular function regulator (Fig. 5b). Finally, the three most abundant classes of the biological processes were the cellular process, single-organism process, and metabolic process (Fig. 5c). Functional annotation revealed that 56 proteins were involved in angiogenesis, as presented in Table 2.

**Table 2:** Protein identified in fat extract related to angiogenesis

Protein name	Gene name	Protein name	Gene name
Myeloid-derived growth factor	MYDGF	Focal adhesion kinase 1	PTK2
Integrin alpha-V	ITGAV	Lysosomal Pro-X carboxypeptidase	PRCP
Basement membrane-specific heparan sulfate proteoglycan core protein	HSPG2	Transforming protein RhoA	RHOA
Heat shock protein beta-1	HSPB1	Collagen alpha-1(XVIII) chain	COL18A1
Ras-related protein R-Ras	RRAS	Protein PML	PML
Heat shock protein beta-6	HSPB6	Programmed cell death protein 6	PDCD6
Neuropilin-1	NRP1	Annexin A2	ANXA2
Tryptophan—tRNA ligase, cytoplasmic	WARS	Cell surface glycoprotein MUC18	MCAM
Decorin	DCN	SPARC	SPARC
Integrin beta-1	ITGB1	Ribonuclease inhibitor	RNH1
Thymidine phosphorylase	TYMP	Collagen alpha-2(IV) chain	COL4A2
Histidine-rich glycoprotein	HRG	1-Phosphatidylinositol 4,5-bisphosphate phosphodiesterase delta-1	PLCD1
Alpha-parvin	PARVA	Aminopeptidase N	ANPEP
*N*(G),*N*(G)-dimethylarginine dimethylaminohydrolase 1	DDAH1	GDP-fucose protein *O*-fucosyltransferase 1	POFUT1
Chloride intracellular channel protein 4	CLIC4	General transcription factor II-I	GTF2I
Caveolin-1	CAV1	Serine/threonine-protein phosphatase 2B catalytic subunit beta isoform	PPP3CB
ATP synthase subunit beta, mitochondrial	ATP5F1B	Thy-1 membrane glycoprotein	THY1
Nucleolin	NCL	Collagen alpha-1(IV) chain	COL4A1
Cell division control protein 42 homolog	CDC42	Chondroitin sulfate proteoglycan 4	CSPG4
Myosin-9	MYH9	Annexin A3	ANXA3
Aquaporin-1	AQP1	Glutathione peroxidase 1	GPX1
Signal transducer and activator of transcription 1-alpha/beta	STAT1	Chymase	CMA1
Fibronectin	FN1	Glucose-6-phosphate isomerase	GPI
Urotensin-2	UTS2	Cadherin-13	CDH13
Calcineurin subunit B type 1	PPP3R1	Apolipoprotein D	APOD
Aminoacyl tRNA synthase complex-interacting multifunctional protein 1	AIMP1	Endoplasmic reticulum aminopeptidase 1	ERAP1
Collagen alpha-1(XV) chain	COL15A1	Transmembrane glycoprotein NMB	GPNMB
Receptor-type tyrosine-protein phosphatase mu	PTPRM	Transforming growth factor-beta-induced protein ig-h3	TGFBI

### FE promoted HUVEC proliferation and migration

**Fig. 6:**
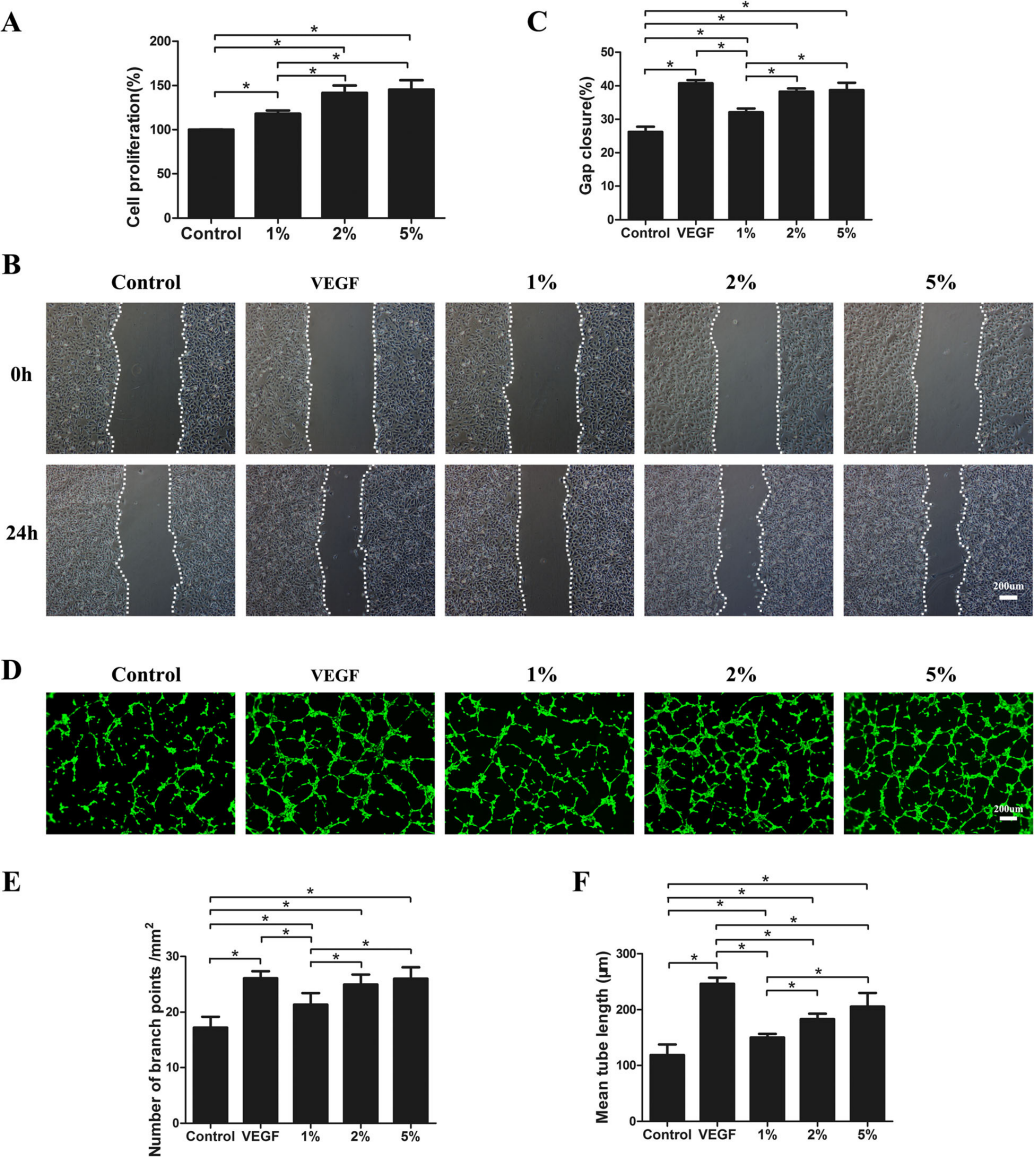
FE promotes endothelial cell proliferation, migration, and tube formation. **a** HUVECs treated with FE at indicated concentrations. Cell proliferation assessed using cell counting kit, and percentage of optical density values relative to control calculated. **b** HUVEC migration evaluated using cell migration assay. **c** Percentage of gap closure (24 h) quantified. **d** HUVECs added to solidified Matrigel in a serum-free medium in presence or absence of FE. VEGF 20 ng/ml used as positive control. After 6-h incubation, endothelial cell tube formation stained using Calcein-AM and assessed by fluorescence microscopy. **e** Assessment of number of branch points/mm^2^ in each group. **f** Quantification of mean tube length. *p < 0.05. VEGF vascular endothelial growth factor

To prove the proangiogenic capacity of FE, the effects of FE on vascular endothelial cell growth and migration were investigated in vitro. HUVECs were treated with various concentrations of FE (0, 1, 2, and 5%) for 3 days. The CCK-8 assay showed that FE promoted HUVEC proliferation in a dose-dependent manner (Fig. 6a). To analyze whether FE could affect HUVEC migration, a wound-healing assay was performed. As shown in Fig. 6b, c, after incubation with different concentrations of FE (0, 1, 2, and 5%) for 24 h, FE enhanced HUVEC migration in a dose-dependent manner. Significant cell proliferation and migration was observed even at a low concentration (1%) of FE treatment.

### FE improved HUVEC tube formation in vitro and promoted vascular formation in vivo as detected using the Matrigel assay

**Fig. 7:**
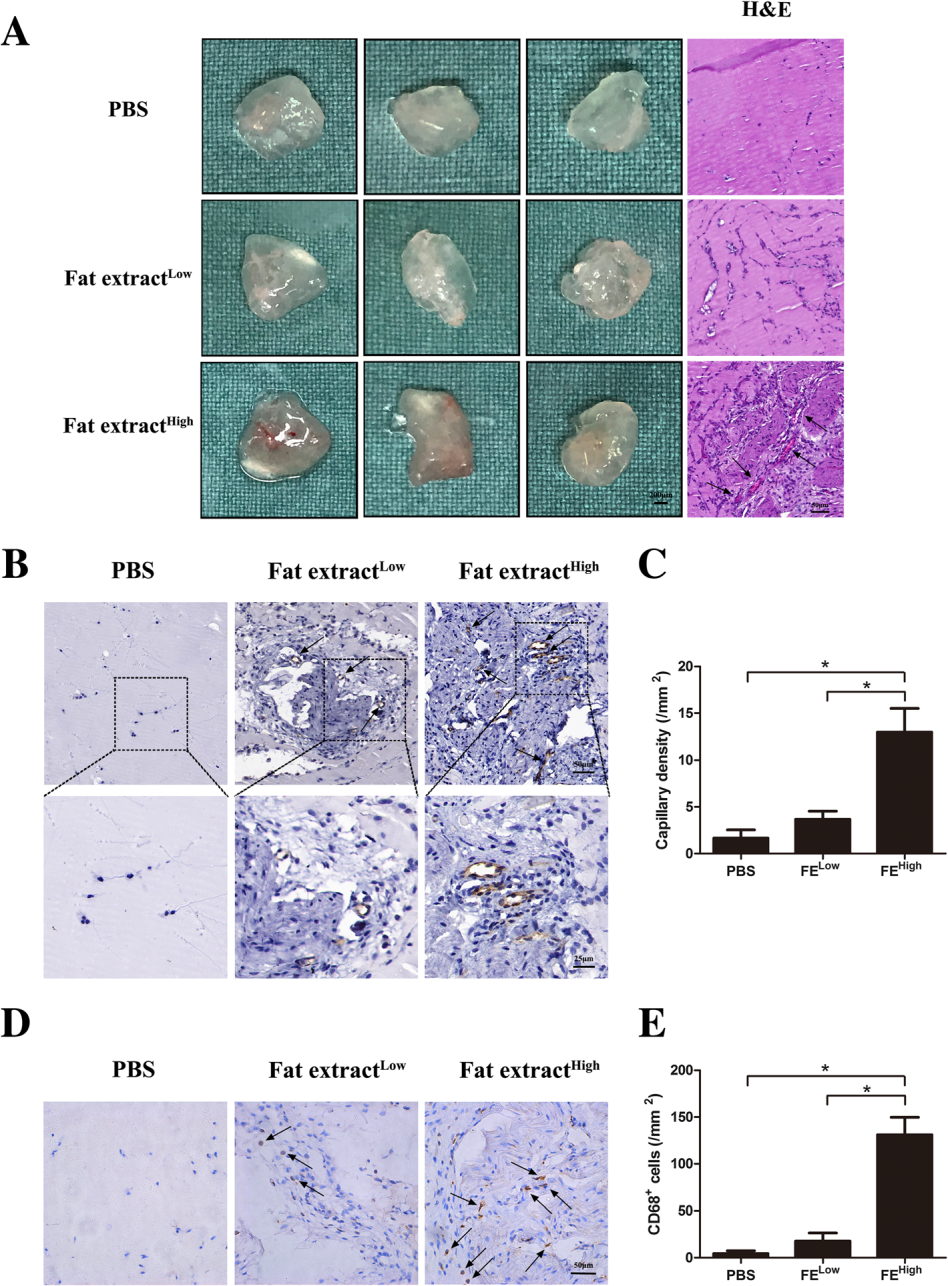
Matrigel plug assay shows that FE promotes angiogenesis in vivo. **a** PBS and FE of low and high concentrations mixed with Matrigel and injected subcutaneously into dorsal region of nude mice. Matrigel plugs harvested at 1 week post implantation. Left panel: gross morphology of Matrigel plugs. Right panel: hematoxylin–eosin (H&E) staining of paraffin sections of explanted plugs. Tissue with more blood vessels observed in FE^High^ plugs. Arrows indicate formed blood vessels. **b** Immunostaining of Matrigel plug with CD31 antibody. FE^High^ group showed more CD31^+^ blood vessels. **c** Quantification of CD31^+^ capillary density. Significantly higher blood vessel density measured in FE^High^-treated group. **d** Immunostaining of mouse macrophage marker CD68 on Matrigel plugs. FE^High^ group showed more CD68^+^ cells. **e** Quantification of CD68^+^ cells. Significantly higher number of CD68^+^ cells observed in FE^High^-treated group. *p < 0.05. PBS phosphate-buffered saline

To further confirm the proangiogenic effects of FE in vitro, a tube formation assay was performed in which HUVECs were treated with different concentrations of FE (0, 1, 2, and 5%) for 6 h. More tubular structures were observed in the FE-treated groups (Fig. 6d); this finding was confirmed by calculating the number of branch points/mm^2^ and measuring the mean tube length in each group (Fig. 6e, f). To study the proangiogenic potential of FE in vivo, the Matrigel plug assay was performed in the nude mouse by subcutaneously injecting the Matrigel mixture, which included different concentrations of FE. The gross view of the plugs harvested after 7 days of implantation showed that reddish grafts were observed in the FE^High^ group, while nearly transparent grafts were observed in the FE^Low^ group and the PBS group (Fig. 7a). Histological analyses via HE staining showed that more tissues with blood vessels were observed in the FE^High^ plugs; less tissues were observed in the FE^Low^ plugs, and almost no tissues were observed in the control plugs (Fig. 7a). Capillary density was measured using immunohistochemical staining and showed that the number of CD31^+^ vascular structures was significantly higher in the FE^High^ group than those in the FE^Low^ and control groups (Figs. 7b, c). Identification of inflammatory cells via immunostaining of mouse macrophage marker CD68 showed significantly higher CD68^+^ cells in the FE^High^ group than those in the FE^Low^ and control groups (Fig. 7d, e), in parallel with the massive cellular infiltration and higher capillary density observed in plugs containing a high dose of FE. Taken together, these results suggest that FE can promote angiogenesis in vitro and in vivo.

## Discussion

Adipose tissues, as well as their derivatives, including nanofat, ADSCs, SVF, and SVF-gel, have been used in the treatment of many ischemic disorders in different animal models [1, 29, 30, 32, 35–37]. The protective effects of those materials are believed to be mainly related to their cellular components via paracrine growth factors. In the current study, we demonstrated for the first time that a cell-free extract from human adipose tissue also possessed powerful therapeutic effects. The extract could significantly attenuate tissue necrosis in a murine limb ischemia model by promoting angiogenesis (Figs. 2 and 3). High levels of various growth factors were detected in FE (Fig. 4). Proteomic analysis revealed that among 1767 proteins detected, 56 were angiogenesis related. The proangiogenic activity was further demonstrated by the enhanced proliferative, migratory, and tube formation ability of HUVECs in vitro when treated with FE (Fig. 6). Moreover, FE enhanced vascular formation in the in-vivo mouse subcutaneously implanted with Matrigel (Fig. 7). These results indicate that FE could be a novel bioproduct potentially used for the treatment of ischemic disorders.

The proangiogenic effect of FE in therapeutic neovascularization can be explained by the presence of high levels of various angiogenic growth factors, including VEGF, PDGF, and bFGF, as measured by ELISA (Fig. 4). A combination of growth factors likely induces angiogenesis synergistically and thus generates long-term functional blood vessels [38–41]. The dose-dependent effect on the proliferation, migration, and tube formation ability of HUVECs was shown in vitro after FE treatment, which might be attributed to effects on VEGF, bFGF, and HGF, respectively, together with their cross-talk with other growth factors [42–47]. The in-vivo early angiogenesis response after FE injection in the Matrigel plugs was demonstrated in our study (Fig. 7). It is clear that in-vivo angiogenesis is a complex multistep event that requires the development of endothelial sprouts, their transformation into capillary plexuses, and the maturation of the primitive vascular network [38, 39, 48]. These processes rely on the interplay between various angiogenic growth factors to initiate neovascularization and remodeling, which may account for the early angiogenesis observed in Matrigel plugs infiltrated with FE. Interestingly, infiltration of CD31^–^ cells was also observed in FE-treated plugs (Fig. 7b). This is possibly due to the presence of multiple growth factors in FE that could recruit different types of cell into the plugs. CD68 staining showed that more CD68^+^ monocytes/macrophages were observed in these samples (Fig. 7d, e), indicating that inflammatory cells may also be involved in the angiogenic process. However, in the limb ischemic model, fewer CD68^+^ cells were observed in the FE-treated groups. This is in accordance with the attenuated necrosis in the ischemic muscle after FE treatment (Fig. 3). Moreover, the marked improvement in blood flow observed in FE-treated ischemic mice at day 28 could be interpreted as the establishment of a stable and functional vascular system. Although we did not compare the therapeutic effects of FE to the effects of using a single recombinant growth factor in this study, we could speculate that FE with a combination of multiple angiogenic factors would achieve better outcomes.

The procedure for generating FE follows the nanofat processing procedure described by Tonnard et al. [20] with some modifications. To obtain a more condensed fat emulsion, we first centrifuged the lipoaspirate before emulsification to remove the watery content. After the mechanical emulsification, the emulsified fat then underwent a freeze/thaw cycle to further lyse the tissues/cells. This freeze/thaw procedure makes it easier to separate the liquid portion from oil droplets and enhances (approximately 30%) the FE yield, but does not change the concentration of growth factors within the FE (data not shown). Thus, this procedure could be omitted for the urgent use of FE in the clinical setting. The final procedure consisted of passing the extract through a 0.22-μm filter to remove the residual cells and cell debris as well as the unexpected contaminating bacteria during processing, which resulted in a pure cell-free, bacteria-free extract that could be safely used in the clinical setting. The whole procedure is relatively simple and safe, as only physical methods were used to break adipose tissue and separate components; no enzymes and chemical reagents were involved during the entire process. For those who later may need multiple injections, cryopreserved FE at − 20 °C did not compromise the proangiogenic effects when used within 6 months, which was evaluated in this study. In addition, no cryoprotectant is required during storage. However, the long-term preservation of FE requires future investigations. In the current study, human liposuction aspirates were obtained from healthy female donors aged 24–36 years who underwent liposuction. Unfortunately, we have not measured the body mass index of each donor. It is possible that the composition of FE maybe different from individuals with different age, gender, and body mass index. It is worth comparing this composition in future studies.

Compared with adipose tissues and their derivatives, being cell free is a unique characteristic of FE, which brings a number of advantages during clinical application. Several major challenges involving the use of viable cells could be easily overcome by using FE. For instance, it is difficult to achieve stable and predictable therapeutic effects by using viable cells due to the variation of cell activities from passage to passage. However, by measuring the level of growth factors in FE, it is easy to control the quality of FE, which could eventually control the therapeutic effects. Under pathological conditions, low survival of implanted cells would reduce the therapeutic effects. Clearly, there is no such worry when using FE in pathological conditions. The tumorigenic concern of using viable cells is currently the biggest obstacle behind the use of cell-based therapy clinically. No such issue exists in the use of cell-free FE. More importantly, immune rejection commonly occurs when using allogeneic cells. Theoretically, no immune rejection would occur when using cell-free FE, suggesting that FE could be a potential “off-the-shelf” product for the treatment of ischemic disorders.

Over the past decade, efforts in using adipose tissue-mediated regenerative therapies have focused on ADSCs. Mechanistic studies of ADSC-based therapy have shown that paracrine secretion is responsible for its therapeutic efficacy. The beneficial paracrine effects of ADSCs have been demonstrated in multiple clinical trials and basic studies involving angiogenesis, immunomodulation, tissue regeneration, etc. [49–52]. Our preliminary study found that the secretion profile of ADSCs is similar to that of FE, but the levels of most factors are substantially lower (data not shown). Obviously, FE is different from the secretome of ADSCs. The mechanical process of the lipoaspirate breaks down most mature adipocytes as well as many other cell types, including stromal cells, endothelial cells, and blood cells. Proteomic analysis of FE from three samples showed that 1767 proteins were identified (Fig. 5). Among these, 56 proteins were angiogenesis related (Table 2). The major proangiogenic components in FE are under investigation. In addition, the proteomic profiles of FE and ADSC secretome, as well as the therapeutic effects of both materials in different disease models, are worthy of comparison in future studies. It cannot be denied that the function of FE is transient, while the transplantation of viable cells could continuously release growth factors. Therefore, a multiple-dosing strategy is recommended in future clinical practice. The therapeutic effects of FE have also been achieved in other ischemic models, including improving skin flap survival, fat transplantation, and stroke recovery (unpublished data).

In addition to the proangiogenic contribution described in the present study, FE may hold great promise for other regenerative purposes due to the presence of various multipotent growth factors. For example, the high concentration of neurotrophic factors within FE may indicate its potential efficacy in the regeneration of nervous tissue; the enrichment of HGF suggests that FE may be useful for treating liver damage; and the presence of EGF may suggest its possible effects on wound healing. Other functional roles of FE are worthy of exploration in future studies.

## Conclusions

We developed a novel cell-free therapeutic agent, FE, which was produced from human adipose tissue through simple physical methods. FE was able to attenuate ischemic injury in a mouse hindlimb ischemic model through its ability to promote angiogenesis. FE could be a novel cell-free therapeutic agent in the treatment of ischemic disorders.

## Abbreviations

ADSC: Adipose tissue-derived stromal/stem cell
BDNF: Brain-derived neurotrophic factor
bFGF: Basic fibroblast growth factor
CM: Conditioned medium
EGF: Epidermal growth factor
ELISA: Enzyme-linked immunosorbent assays
FE: Fat extract
G-CSF: Granulocyte colony-stimulating factor
GDNF: Glial cell-derived neurotrophic factor
GM-CSF: Granulocyte–macrophage colony-stimulating factor
HGF: Hepatocyte growth factor
HUVEC: Human umbilical vein endothelial cell
IGF-1: Insulin-like growth factor 1
IL-6: Interleukin 6
NT-3: Neurotrophin-3
PDGF: Platelet-derived growth factor
SVF: Stromal vascular fraction
TGF-α: Transforming growth factor alpha
TGF-β: Transforming growth factor beta
VEGF: Vascular endothelial growth factor
